# The Role of the Suprachiasmatic Nucleus in Cardiac Autonomic Control during Sleep

**DOI:** 10.1371/journal.pone.0152390

**Published:** 2016-03-24

**Authors:** S. D. Joustra, R. H. Reijntjes, A. M. Pereira, G. J. Lammers, N. R. Biermasz, R. D. Thijs

**Affiliations:** 1 Department of Medicine, Division of Endocrinology, Centre for Endocrine Tumours Leiden, Leiden University Medical Centre, Leiden, Netherlands; 2 Department of Neurology, Leiden University Medical Centre, Leiden, Netherlands; 3 Stichting Epilepsie Instellingen Nederland (SEIN), Heemstede, Netherlands; Oasi Institute for Research and Prevention of Mental Retardation, ITALY

## Abstract

**Background:**

The suprachiasmatic nucleus (SCN) may play an important role in central autonomic control, since its projections connect to (para)sympathetic relay stations in the brainstem and spinal cord. The cardiac autonomic modifications during nighttime may therefore not only result from direct effects of the sleep-related changes in the central autonomic network, but also from endogenous circadian factors as directed by the SCN. To explore the influence of the SCN on autonomic fluctuations during nighttime, we studied heart rate and its variability (HRV) in a clinical model of SCN damage.

**Methods:**

Fifteen patients in follow-up after surgical treatment for nonfunctioning pituitary macroadenoma (NFMA) compressing the optic chiasm (8 females, 26–65 years old) and fifteen age-matched healthy controls (5 females, 30–63 years) underwent overnight ambulatory polysomnography. Eleven patients had hypopituitarism and received adequate replacement therapy. HRV was calculated for each 30-second epoch and corrected for sleep stage, arousals, and gender using mixed effect regression models.

**Results:**

Compared to controls, patients spent more time awake after sleep onset and in NREM1-sleep, and less in REM-sleep. Heart rate, low (LF) and high frequency (HF) power components and the LF/HF ratio across sleep stages were not significantly different between groups.

**Conclusions:**

These findings suggest that the SCN does not play a dominant role in cardiac autonomic control during sleep.

## Introduction

Sleep exerts major effects on cardiac autonomic control. For example, compared to slow-wave sleep, rapid eye movement sleep is associated with increased heart rate (HR) and low-frequency power of HR variability (HRV), and decreased high-frequency power [1]. The suprachiasmatic nucleus (SCN) is the critical relay of the diurnal sleep-wake regulation [2]. It may also play an important role in central autonomic control, as tracing studies demonstrated that SCN neurons project to the paraventricular nucleus and connect with parasympathetic and sympathetic relay stations in the brainstem and spinal cord [3,4]. Accordingly, SCN lesions in rat reduced the HR decrease during resting periods [5]. Furthermore, humans showed diurnal rhythms of HR and HRV independent of sleep stage in a constant routine protocol [6]. Consequently, it may well be argued that cardiac autonomic control during nighttime not only results from direct effects of sleep-related changes in the central autonomic network, but also from endogenous circadian factors as directed by the SCN.

Nonfunctioning pituitary macroadenomas (NFMA) have been proposed as a model of SCN damage [7], and thus provide an unique opportunity to study the influence of the SCN on autonomic fluctuations during sleep in humans. NFMA compress surrounding tissue, causing hypopituitarism and/or visual impairments. Transsphenoidal adenomectomy, sometimes complemented with radiotherapy, usually improves visual function, but hypopituitarism may persist [8]. Additionally, long-term remission is accompanied by poor subjective sleep quality, fragmented sleep-wake patterns, and alterations of diurnal melatonin and temperature rhythmicity [9,10]. These symptoms were strongly associated with suprasellar tumour extension (irrespective of hypopituitarism), implying damage to the adjacent SCN. To understand the role of the SCN in cardiac autonomic control during sleep, we studied HR control during nighttime in patients treated for NFMA and age-matched controls.

## Methods

### Participants

Seventeen adult patients surgically treated for NFMA and seventeen age-matched healthy controls underwent a single night of ambulatory polysomnography [9]. All patients were otherwise healthy and received yearly follow-up by an endocrinologist. Exclusion criteria were age > 65 years, use of psychotropic or cardiac medication, diagnosis of a sleep disorder, hypertension, dyslipidemia, or diabetes mellitus. HRV data from two patients and two controls were excluded for insufficient data quality for this analysis (detached HR electrode), thus fifteen patients and fifteen healthy controls were studied.

Markers of circadian rhythmicity in these patients and their methods were previously published [9,10], and included intradaily variability, skin temperature, and melatonin secretion profiles. The intradaily variability, derived from 7 days of actigraphy, quantifies how fragmented the rhythm of motor activity is relative to its 24-h amplitude; more frequent alterations between an active and an inactive state lead to a higher intradaily variability [11]. Proximal skin temperature was measured for 24 hours at both infraclavicular areas, supra-umbilical, and on the left mid-thigh. Its diurnal variation is related to increased sleep latency in both narcoleptics and healthy persons [12,13], and to sleep depth [14]. In our previous study differences in proximal skin temperature between NFMA patients and controls were more pronounced than those in distal skin temperature or core body temperature [10]. Melatonin secretion was measured for 36 hours using at least thirteen saliva samples. An altered melatonin profile was defined as the absence of an evening rise or daytime values >3 pg/mL in a 36-hour salivary melatonin collection. We refer to S1 Table for the values of these markers for each patient.

The ethical committee of the Leiden University Medical Center approved the study. All subjects gave written informed consent.

### HRV

Methods of polysomnography [9] and calculation of HRV [15,16] have been detailed previously. In short, sleep stages, apnea/hypopnea events and leg movements were manually scored in 30-second epochs by an experienced sleep technician. All HR data from nocturnal sleep onset until morning awakening were selected. A continuous wavelet transform was implemented in Matlab (Version 13.1, Mathworks, MA, USA) to detect R-peaks in the 30-second epochs. Outliers, differing > 25 beats per minute from adjacent samples, were excluded. Heart beat data were resampled at 5 samples per second. The full spectrum of HRV in the region surrounding each sample was calculated with a fast Fourier transform using a window of 512 samples, ranging 256 samples left and right of a particular sample. After averaging these spectra for each 30-second epoch, the LF (0.04–0.15 Hz) and HF (0.15–0.4 Hz) power components per epoch were calculated. The LF band is thought to predominantly reflect the baroreflex-mediated sympathetic activity [16] whereas the HF band is thought to represent an index of vagal activity, and the LF/HF ratio an index of the sympathovagal balance [17]. All measures have their limitation and should be interpreted cautiously. To account for the autonomic effects of arousals, we identified all epochs with apnea, hypopnea, leg movements, or transitions from NREM3 to NREM1/2 or NREM2 to NREM1. The raw data file on HR and HRV is available in the S1 Data File.

### Statistical analysis

Patients’ clinical characteristics were compared with controls using the Student’s *t*-test for numerical data (or in case the assumption of normality was not met (Shapiro-Wilk test) the Mann-Whitney U test), and Pearson’s χ^2^ for categorical data. Mixed effect regression models were used to analyse the effect of disease on HR, LF, HF, and LF/HF. This model included all 30 second epochs. Epochs were classified as rapid eye movement- (REM), non-REM stage 1- (NREM1-), NREM2-, and NREM3-sleep. Disease, wake and sleep stages, leg movements, apnea events, gender, and arousal transitions were selected as fixed effects and participants as random effect. A natural logarithm-transformation was applied to obtain a normal distribution of the model’s residuals. Differences were considered statistically significant at *P* < 0.05. In a secondary analysis, the interaction between disease and each of the sleep stages was added to the model, to explore sleep stage-dependent differences in HRV. To correct for multiple testing after introducing the interaction terms (five sleep stages), sleep stage-dependent differences were considered statistically significant at *P* < 0.01.

## Results

### Participants

Median age was 58 yr (range 26–65 yr) for NFMA patients (8 females), and 52 yr (range 30–63 yr) for the control subjects (5 females) (Table 1). Prior to surgery, most patients had visual field defects (80%) or suprasellar extension on MRI (93%). Hypopituitarism was present in eleven (deficiency of growth hormone in ten, of corticotropin in five, of thyrotropin in ten, and of the gonadotropins in five); all received hormone replacement, except for optional growth hormone replacement, which was left untreated in three patients. Healthy control subjects were not significantly different in terms of gender, age, or BMI. Compared to controls, patients spent more time awake (*P* = 0.005) and in NREM1-sleep (*P* < 0.001), and less in REM-sleep (*P* < 0.001). Mean duration of apnea was shorter in patients (13.4 ± 9.1 seconds *vs*. 21.8 ± 8.0 seconds, *P* = 0.008).

**Table 1:** Clinical characteristics of patients with NFMA and healthy age-matched control subjects.

Parameter	NFMA	Controls	*P*-value
*N*	15	15	
No. of females	8 (53%)	5 (33%)	0.269
Age, median (range)(yr)	58 (26–65)	52 (30–63)	0.220
BMI (kg/m^2^)	27.3 ± 4.1	25.3 ± 2.5	0.125
Years after surgery	6 (1–18)		
Adjuvant radiotherapy	5 (33%)		
VFD at presentation	12 (80%)		
Suprasellar extension	14 (93%)		
Pituitary insufficiency	11 (73%)		
Intradaily variability of activity^‡^^a^	0.43 ± 0.09	0.35 ± 0.08	**0.014**
Altered melatonin profile^‡^^b^	5 (33%)	0 (0%)	**0.042**
Proximal skin temperature daytime^‡^^c^	33.5 ± 0.6	33.9 ± 0.4	**0.039**
Sleep characteristics^d^			
Sleep period (minutes)	499.1 ± 92.7	477.5 ± 56.6	0.373
Total sleep time (minutes)	438.6 ± 101.5	448.0 ± 61.0	0.852
Sleep efficiency (%)	87.1 ± 8.2	93.1 ± 3.1	**0.010**
% non-REM 1 of TST	20.2 ± 10.3	9.6 ± 4.3	**<0.001**
% non-REM 2 of TST	41.4 ± 9.2	41.4 ± 6.7	0.989
% non-REM 3 of TST	20.9 ± 9.4	23.4 ± 5.3	0.379
% REM of TST	17.4 ± 5.2	25.5 ± 4.3	**<0.001**
% WASO	12.8 ± 8.1	6.3 ± 3.1	**0.005**
PLM per hour	18.9 ± 21.8	16.4 ± 7.3	0.296
Apnea-hypopnea index	3.7 ± 5.2	6.8 ± 6.2	0.074
Mean apnea duration (seconds)	13.4 ± 9.1	21.8 ± 8.0	**0.008**
Desaturation periods^e^ (%)	0.2 (0.0–9.6)	0.0 (0.0–0.9)	0.075

Data represent number (percentage), average ± SD, or median (P10-P90), unless described otherwise.

^‡^These results were previously published [9, 10].

^a^From 7 days of actigraphy recording [9].

^b^Absence of evening rise or daytime values >3 pg/mL in a 36-hour salivary melatonin collection [10].

^c^From 24-hour skin temperature measurements [10].

^d^Assessed with polysomnography.

^e^Percentage of total sleep time in which saturation was below 90%.

VFD, Visual field defects; TST, total sleep time; WASO, wake after sleep onset; PLM, period limb movements. Statistically significant differences are marked in bold.

### HRV

**Fig 1:**
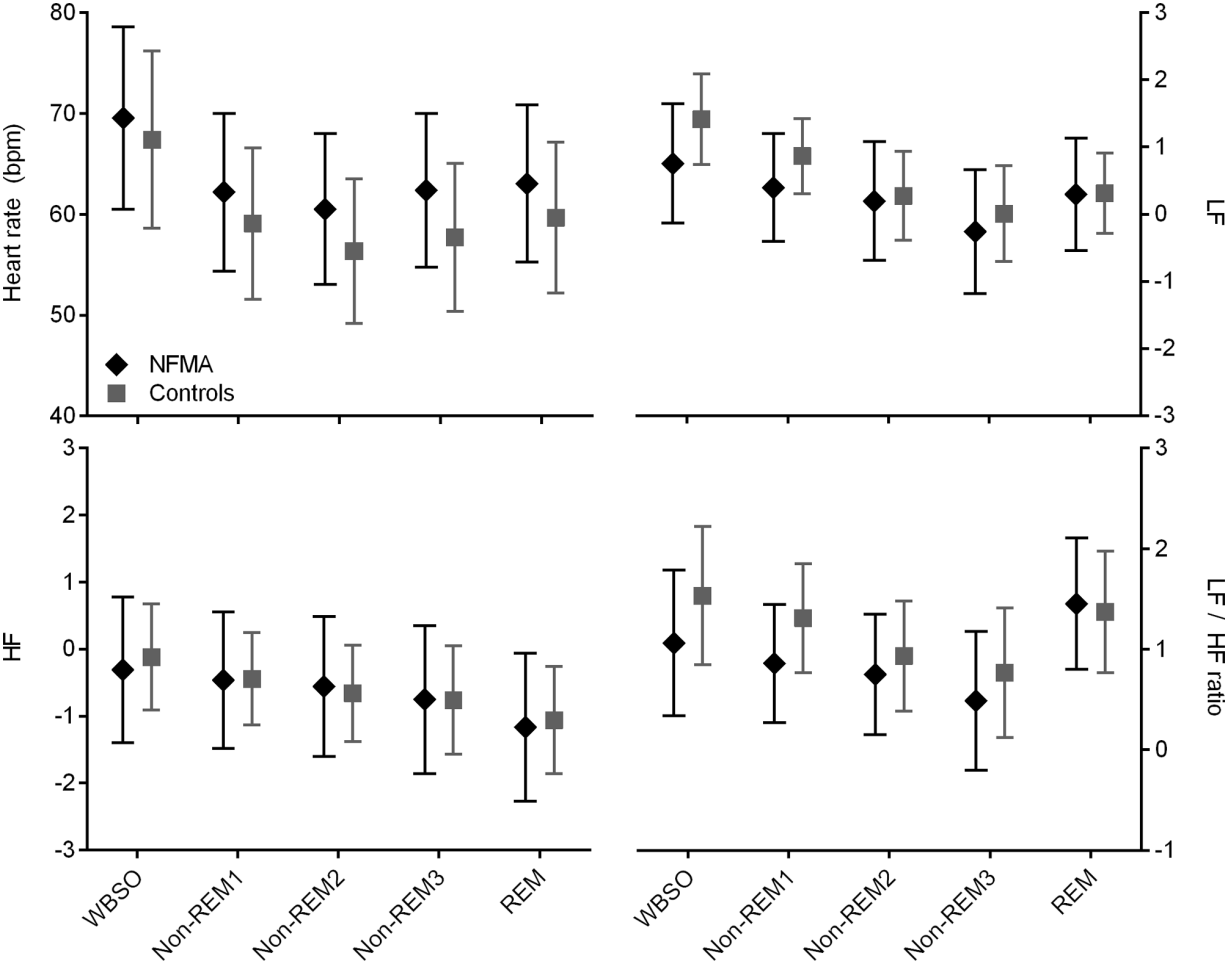
Heart rate variability in patients and controls. Heart rate, low- and high frequency power and their ratio, in 15 NFMA patients and 15 healthy controls, stratified for wake and sleep stages. Data represent mean ± SD, calculated over averages for each subject. WBSO, wake before sleep onset; REM, rapid eye movement sleep; LF, low frequency power in ln(ms^2^/Hz); HF, high frequency power in ln(ms^2^/Hz). None of the differences reached statistical significance after correction for multiple testing (*P* < 0.01) in our secondary analysis.

We included an average total number of 1374 epochs per participant in our model. Patients did not differ from their age-matched controls during the night with respect to HR (*P* = 0.137), LF (*P* = 0.205), HF (*P* = 0.959), or LF/HF (*P* = 0.184) (Table 2). As differences might be dependent on patients with the most prominent signs of circadian dysrhythmicity, *e*.*g*. those with disturbed melatonin secretion, this feature was added as an additional coefficient to the model, but lacked significance. Fig 1 displays the results from the secondary analysis, exploring sleep stage—dependent differences in HR and HRV between patients and controls. LF/HF was slightly lower in patients than controls (difference: -0.44 [standard error: 0.20], *P* = 0.040), but this difference did not reach statistical significance after correction for multiple testing (*P* > 0.01).

**Table 2:** Effects of mixed effects regression model coefficients on heart rate and heart rate variability.

Parameter	HR	LF	HF	LF/HF
Intercept^1^	73.60 ± 2.73***	1.17 ± 0.21***	0.43 ± 0.28	1.11 ± 0.19***
Non-REM1	-13.02 ± 1.03***	-0.39 ± 0.10***	-0.61 ± 0.11	-0.45 ± 0.09***
Non-REM2	-15.12 ± 0.94***	-0.72 ± 0.12***	-0.59 ± 0.11	-0.67 ± 0.08***
Non-REM3	-13.42 ± 0.98***	-1.06 ± 0.13***	-0.21 ± 0.13	-0.85 ± 0.10***
REM	-12.07 ± 1.02***	-0.63 ± 0.11***	-0.55 ± 0.13***	0.08 ± 0.10
Apnea/hypopnea	-0.34 ± 0.17*	0.46 ± 0.03***	0.27 ± 0.03***	0.19 ± 0.03***
Leg movements	0.97 ± 0.14***	0.78 ± 0.02***	0.06 ± 0.02*	0.73 ± 0.02***
Arousal transitions^2^	2.25 ± 0.20***	0.34 ± 0.03***	0.07 ± 0.03*	0.27 ± 0.03***
Gender	-4.09 ± 2.73	-0.38 ± 0.22	-1.11 ± 0.29***	0.70 ± 0.19**
Disease	4.19 ± 2.70	-0.22 ± 0.23	-0.03 ± 0.31	-0.20 ± 0.19

Values represent the effect of model coefficients on HR, LF, HF, and LF/HF, displayed as the mean (± SEM) deviation from the intercept (*i*.*e*. wake before sleep onset). HR, heart rate in beats per minute; LF, low frequency power in ln(ms^2^/Hz); HF, high frequency power in ln(ms^2^/Hz); REM, rapid eye movement sleep.

^1^Wake before sleep onset.

^2^Transition from deep to light sleep.

*P < 0.05;

**P < 0.01;

***P < 0.001.

## Discussion

We studied the role of the SCN in sleep-related cardiac autonomic control in a cohort of NFMA patients and healthy age-matched controls and did not identify major autonomic alterations. Our results suggest that modulation of sleep-related cardiac autonomic control is predominantly linked to sleep processes with a subordinate role for the SCN. This is supported by experiments of shifting the sleep period, inducing a concomitant shift in the diurnal variation of HRV [18], in contrast to other circadian parameters such as the melatonin peak and core body temperature nadir that showed respectively no shift and a biphasic curve in similar experiments [19]. Furthermore, preservation of the effects of sleep on HR in patients with bilateral carotid tumour resection also suggests that these alterations are predominantly generated through central, non-baroreflex mediated pathways [16]. In rats a bidirectional relationship has been demonstrated between sleep processes and SCN activity [20]. Sleep processes might therefore influence cardiac control through altering SCN activity. In view of our findings it seems however likely that other central circuitries linking sleep and cardiac autonomic control, *e*.*g*. the hypothalamic nuclei [21, 22], outweigh this influence.

Our study has limitations. First, the extent of SCN dysfunctioning in our patients is not exactly known, since at present no parameter is available to directly measure SCN functioning. However, all patients had indirect signs of SCN dysfunctioning, *e*.*g*. altered rhythmicity of melatonin/temperature/sleep, albeit in variable patterns [9,10]. Second, the sample size limits the statistical strength to rule out small differences. It should however be noted that even smaller sample sizes were able to demonstrate distinct and consistent autonomic differences across sleep stages in narcolepsy and bilateral carotid body resection, using identical study protocols [15,16].

In conclusion, we did not observe major differences in HR and HRV between NFMA patients and controls during sleep. The findings suggest that the SCN does not play a dominant role in cardiac autonomic control during sleep.

## Supporting Information

S1 Data File Original HR and HRV analysis results. (XLSX)

S1 Table Individual values of markers of circadian rhythmicity. (DOCX)
