# Supplementary material for: The Role of the Suprachiasmatic Nucleus in Cardiac Autonomic Control during Sleep
Source: PLoS One. 2016 Mar 24;11(3):e0152390. doi: 10.1371/journal.pone.0152390 (PMC4807027; doi:10.1371/journal.pone.0152390)
Supplement: S1 Table — (DOCX) [file pone.0152390.s002.docx]

**S1 Table.** Individual values of markers of circadian rhythmicity

| Patient ID | Intradaily  Variability^a^ | Proximal skin  temperature (ᵒC)^b^ | Melatonin secretion profile^c^ |
| --- | --- | --- | --- |
| 1 | 0.46 | 33.2 | No evening rise |
| 2 | 0.51 | 34.3 | No evening rise |
| 3 | 0.37 | 32.8 | *Normal* |
| 4 | 0.32 | 33.7 | *Normal* |
| 5 | 0.61 | 34.0 | Daytime values > 3 pg/mL |
| 6 | 0.37 | 34.3 | *Normal* |
| 7 | 0.35 | 33.8 | *Normal* |
| 8 | 0.40 | 33.0 | *Normal* |
| 9 | 0.49 | 33.7 | *Normal* |
| 10 | 0.37 | 32.2 | *Normal* |
| 11 | 0.45 | 33.0 | Daytime values > 3 pg/mL |
| 12 | 0.41 | 33.6 | *Normal* |
| 13 | 0.53 | 33.8 | *Normal* |
| 14 | 0.30 | 33.8 | No evening rise |
| 15 | 0.49 | 33.4 | *Normal* |

^a^Controls: 0.35 ± 0.08

^b^Controls: 33.9 ± 0.4 ᵒC

^c^Controls: all normal
